# ADVANCING DIVERSITY IN AGING: THE AGE-ADAR SCHOLARS PROGRAM AT WVU

**DOI:** 10.1093/geroni/igad104.0371

**Published:** 2023-12-21

**Authors:** Julie Hicks Patrick, Amber Rusch, Carly Pullen

**Affiliations:** West Virginia University, Morgantown, West Virginia, United States; West Virginia University, Morgantown, West Virginia, United States; West Virginia University, Morgantown, West Virginia, United States

## Abstract

As the number and racial/ethnic diversity of older Americans, ages 65+ years increases, recruiting, training, and retaining a diverse workforce to support aging-related fields is essential. Supported by an R25 grant from the NIA, the goals of the Appalachian Gerontology Experiences: Advancing Diversity in Aging Research (AGE-ADAR) Scholars Program include training undergraduate MSTEM majors in aging and health disparities research, especially as these topics interact in Appalachia. In this brief presentation, we will review the goals and mechanisms through which these aims are accomplished. We will review both hard outcomes (i.e., conference presentations, publications) and soft outcomes (attitude change, career specialization) among our first two cohorts of Scholars. For example, each Scholar prepares two empirical abstracts, presents a poster on campus, presents one or more posters off-campus, produces a public-facing infographic about health disparities and aging, conducts a literature review, and composes an integrated essay related to how their MSTEM major can help to solve issues of health disparities among older adults in Appalachia. About half of our Scholars have co-authored published manuscripts, as well. Finally, other funding mechanisms to support training the next generation of researchers, scholars, and applied workers in aging will be discussed.

